# Impact of Corporate Reputation on Brand Differentiation: An Empirical Study from Iranian Pharmaceutical Companies

**Published:** 2017

**Authors:** Afshin vahabzadeh, Hossein vatanpour, Rasoul dinarvand, Ali rajabzadeh, Jamshid salamzadeh, Mehdi mohammadzadeh

**Affiliations:** a *Department of Pharmacoeconomics and Pharmaceutical Management, School of Pharmacy, Shahid Beheshti University of Medical Sciences, Tehran, Iran. *; b *Department of pharmaceutics, School of pharmacy, Tehran University of Medical Sciences, Tehran, Iran. *; c *Department of Industrial Management, Faculty of Management and Economics, Tarbiat Modarres University, Tehran, Iran.*

**Keywords:** Corporate Reputation, Value Creation, Strategic Resource, Corporate Communication, Brand, Brand Differentiation

## Abstract

The influence of company reputation or what is often referred to as corporate reputation on branding strategy and producing intangible asset for different industries has been researched in western countries, but there is a gap for the generalizability of findings to countries out of the United State and Europe. To establish the western researcher’s external validity of theories in other countries and to obtain a better understanding of the influences of branding and company reputation on pharmaceutical business markets, the researchers applied this study for Iran, a country in the Middle East. The obtained results using SEM (by P.L.S. 2.0 software) showed a good relationship between value creation and brand differentiation (β =0.360 and t-value = 3.167), between corporate communication and brand differentiation (β = 0.022 and t-value = 3.668), and between strategic resources and brand differentiation (β = 0.289 and t-value = 2.247). This study is a pioneering attempt in Iran to measure the impact of corporate reputation on brand differentiation strategy.

## Introduction

In the pharmaceutical industry, branding strategies such as advertising and academic reports mainly influence a doctor’s perception of a product (1). However, a good recognition of a company also helps the customer to decide better. The information a company sends concerning itself has an influential and unexpected impact on consumers’ perception. The signals sent by a firm through its reputation, advertisements, and products price are usually interpreted differently by their customers. Therefore, understanding the fact that how managers use their corporate reputation to establish a firm’s brand image strategy is necessary (2, 3).

A well-communicated image should contribute to establish a brand position, insulate the brand from competition (4), and therefore enhance the brand market performance (5). This potential impact underscores the importance of managing the image and differentiation of brands over time.

Several researches and studies have related the concept of brand differentiation to a firm’s corporate reputation. Ghose *et al*. (2006) suggested that several dimensions of reputation, including packaging, responding to problems, customer service, delivery and product-specific comments, present the principle points that customers seek in their purchase (6). These dimensions not only provide a basis on which sellers can improve their reputation but also help them differentiate themselves from other competitors.

In addition, Brammer and Pavelin (2006) suggested that, corporate reputation could be augmented by different activities, which are closely related to the vertical differentiation of products, such as cultivation of a strong brand image and technological advancement (7). However, a good corporate reputation can also help to differentiate the brand (8).

Furthermore, Gotsi and Wilson (2001) suggested that nowadays, organizations and companies increasingly recognize customers as their most important assets to build a favorable corporate reputation (9). Through respondents’ interviews of the importance of employees in corporate reputation management, Gotsi and Wilson emphasized that employees, as one factor to enhance a firm’s corporate reputation, can contribute to differentiate an organization from its rivals, since consumers evaluate the corporate reputation behind the brand and products presented to them.

Therefore, many organizations use corporate reputation as an important resource to develop their strategic value or as a signal or trait to forecast their potential behavior.

Pharmaceuticals similar to other organizations are encouraged to develop a good corporate reputation for their quality of products (10, 11), for innovativeness (10, 12, 13), for honest communication, and for environmental responsibilities (10). In turn, these factors can be also converted subconsciously into the brand differentiation of the products belonging to the company (14, 15). Since a firm’s corporate reputation tends to influence the initiation of a brand strategy decisions and brand scene-setting in pharmaceutical companies highly (16), therefore, the purpose of this paper is to develop an integrated model that explicitly accounts for the influences of brand differentiation 

and company reputation on business customers’ perception in the Iranian pharmaceutical manufacturing companies.

*Literature Review*

*Corporate Reputation*

Corporate reputation has become a “hot” topic in the past few years given the evidence linking a favorable corporate reputation and various intangible and tangible benefits (17), while interest in the concept of corporate reputation has gained momentum in the last few years (18). Several definitions purporting to explain the concept of corporate reputation have been offered by various authors (19).

One of the most cited definitions of corporate reputation is proposed by Weigelt and Camerer (1988) in the strategic management perspective. They argued that corporate reputation is an attribute or a set of attributes ascribed to a firm, inferred from the firm’s past actions. It is the belief of market participants regarding a firm’s strategic character (20).

Roberts and Dowling (2002) contend that corporate reputation is the public’s cumulative judgment of firms over time (21).

Some other researchers have discussed corporate reputation as a history of customer perception of the firm, such as collective beliefs that exist in the organizational field concerning a firm’s identity and prominence (22, 23).

Davies (2003) suggests that corporate reputation enables firms to attract customers repeatedly (24).

Ferris (2003) also maintains that positive reputation encourages customers to trust in a seller and Increases their commitment (25).

Therefore, what are the uses and benefits of corporate reputation for different firms and organizations like pharmaceuticals?

A positive corporate reputation offers multiple benefits to a firm, such as the ability to withstand occasional adverse publicity (26), higher levels of customer purchase intention (27), strong organizational identification among employees (28), better attitudes towards companie**s **salespeople and products on the part of industrial purchasers (29), customer loyalty (30), attraction of investors (24), and greater competitive advantage (24, 13).

The review of the oretical literature indicates that the uses of corporate reputation can be theorized along six dominant paradigmatic perspectives, namely: public relations, marketing, management, economic, sociological, and financial_accounting (1, 14), which have a strong overlap in business organizations.

To reduce these overlaps, Chen-Chu Chen (2011) presented a synthesis and categorized the uses of corporate reputation into three groups (1) value creation (a tool for creating value), strategic resources (influencing competitor’s actions/strategies), and corporate communication (developing the relationship with stakeholders).

1_ Value Creation_ The most important study in this field is conducted by Dolphin (2004). He argues that corporate reputation is a value-creating tool (31), which has a positive influence on firms’ value (26, 32, and 33). A similar argument was presented under financial and accounting perspective. Moerman and Laan (2006) maintain that corporate reputation is used and presented to stakeholders as a corporate valuable asset** (**34) or as an intangible asset creating value in the future (35). Similarly, Shkolnikov *et al*. (2004) express that corporate reputation is used as a value-creating mechanism (36).

2_ Strategic Resources_ Many scholars assert that corporate reputation can influence competitor’s actions and strategies; therefore, it can be used as a strategic resource (20, 21).

Fombrun and van Riel (2004) suggest that firms use corporate reputation to create a distinctive position in the mind of stakeholders (37) and thus can attain competitive advantage which is a part of strategic resource (10, 38).

Many scholars also argue that corporate reputation is often deployed by firms as a helpful signal. It provides stakeholders with a good insight into the future of a firm and may be used as a signal that enables key resource providers such as banks and other financial institutions to interpret a company’s initiatives from its past actions and assess its ability to deliver value outcomes. All of these can be used by firms as a strategic resource (39, 40, and 41).

3_ Corporate Communication _ it is one of the corporate reputations uses which develops the relationship with stakeholders including internal and external stakeholders. Lerbinger (1965) and Grunig *et al*. (1992) argue that corporate reputation is used to communicate a firm’s social responsibility activities with stakeholders within the business environment (42, 43).

Stanwick and Stanwick (1998) have also been highly vocal regarding the positioning of corporate reputation, which enhances the generation of better feedback from stakeholders within the business environment (44). Fombrun and Shanly (1990) indicate that a firm’s previous corporate reputation can enhance its future reputation among customers.

Management scholars (45) suggest that a firm’s corporate reputation commonly shapes the opinions as well as perceptions of shareholders and stakeholders. Puente *et al*. (2007) argue that a firm’s corporate reputation signals or enables businesses to predict human behavior in the future (46).

*Brand and Brand Differentiation*

Keller (1998) expresses that a brand is a set of mental associations, held by the consumer, which add to the perceived value of a product or service (47). These associations should be unique (exclusivity), strong (saliency), and positive (desirable).

Kotler, Adam, Brown, and Armstrong (2003) defined brand as a “name, term, sign, symbol or design, or a combination of these, intended to identify the goods or services of one seller or group of sellers to differentiate them from those of competitors” (48). AMA (American Marketing Association) (2008) redefined “brand” as “name, term, design, symbol or any other feature that identifies one seller’s goods or services as distinct from those of the other sellers” (49). The legal term for brand is trademark. A brand may identify one item, a family of items or all items of those sellers. If used for the firm as a whole, the preferred term is trade name”.

**Table 1.** Qualitative questions based on the literature review

**H1**: Value Creation as a dimension of the uses of corporate reputation has a positive impact on a firm’s brand differentiation strategy.	Can you suggest the characteristics of value creation which tend to encourage the setting of brand differentiation strategy? And why? You, as an effective personnel in corporate branding strategy, to which of the above characteristics would you pay more attention or would like to invest more? And why?
**H2**: Corporate Communication as a dimension of the uses of corporate reputation has a positive impact on a firm’s brand differentiation strategy.	Can you suggest the characteristics of corporate communication which tend to encourage the setting of brand segmentation strategy? And why? You, as an effective personnel in corporate branding strategy, to which of the above characteristics would you pay more attention or would like to invest more? And why?
**H3**: Strategic Resources as a dimension of uses of corporate reputation has a positive impact on a firm’s brand differentiation strategy.	Can you suggest the characteristics of strategic resources which tend to encourage the setting of brand differentiation strategy? And why? You, as an effective personnel in corporate branding strategy, to which of the above characteristics would you pay more attention or would like to invest more? And why?

**Table 2:** Confirmatory factor analysis of value creation

	**Variable ( questions )**	**Measurement** **error**	**Factor loading**	**CR**	**AVE**
				0.92	0.63
1	VC1	0.61	0.67		
2	VC2	0.25	0.83		
3	VC3	0.45	0.79		
4	VC4	0.32	0.81		
5	VC5	0.49	0.77		
6	VC6	0.21	0.84		
7	VC7	0.71	0.62		
8	VC8	0.65	0.67		
9	VC9	0.51	0.71		
10	VC10	0.54	0.74		

Cronbach’s Alpha =0.91; the factor loading is a standardized value, indicating *p*≤0.05

**Table 3:** Confirmatory factor analysis of corporate communication

	**Variable ( questions )**	**Measurement** **error**	**Factor loading**	**CR**	**AVE**
				0.89	0.58
1	CC1	0.37	0.83		
2	CC2	0.33	0.85		
3	CC3	0.61	0.69		
4	CC4	0.38	0.79		
5	CC5	0.41	0.77		
6	CC6	0.39	0.81		

Cronbach’s Alpha =0.88; the factor loading is a standardized value, indicating *p*≤0.05

**Table 4.** Confirmatory factor analysis of strategic resources

	**Variable ( questions )**	**Measurement** **error**	**Factor loading**	**CR**	**AVE**
				0.91	0.65
1	SR1	0.21	0.94		
2	SR2	0.34	0.91		
3	SR3	0.29	0.93		
4	SR4	0.54	0.78		
5	SR5	0.48	0.81		
6	SR6	0.36	0.88		
7	SR7	0.49	0.84		

Cronbach’s Alpha =0.93; the factor loading is a standardized value, indicating *p*≤0.05

**Table 5:** Confirmatory factor analysis of brand segmentation

	**Variable ( questions )**	**Measurement** **error**	**Factor loading**	**CR**	**AVE**
				0.90	0.73
1	BS1	0.32	0.83		
2	BS2	0.37	0.81		
3	BS3	0.26	0.88		
4	BS4	0.48	0.71		
5	BS5	0.41	0.79		

Cronbach’s Alpha =0.92; the factor loading is a standardized value, indicating *p*≤0.05

**Table 6.** Summary of the tests and results of hypotheses

Hypotheses	Relationships	Path coefficient	t_ value	Result
H1	VC BD	0.360	3.167	**Accepted**
H2	CC BD	0.022	3.668	**Accepted**
H3	SR BD	0.289	2.247	**Accepted**

T_ values Significant at *P* ≤0.05.

**Figure1:**
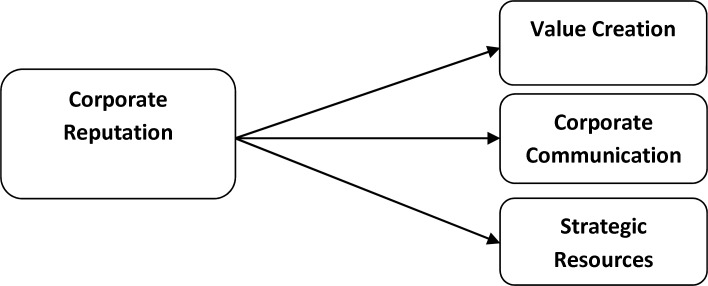
Corporate Reputation and its uses

**Figure 2:**
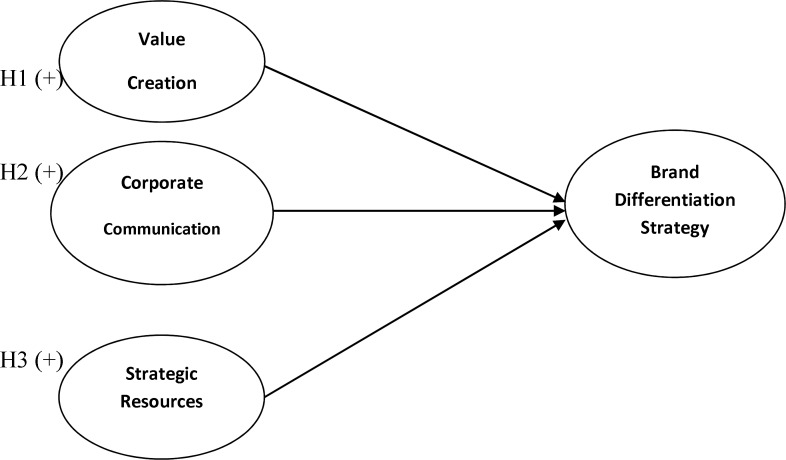
The Conceptual Framework and Hypotheses

**Figure 3:**
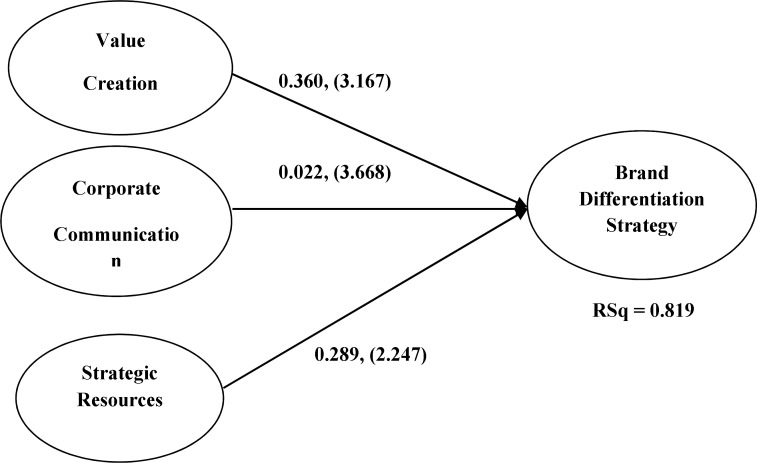
Path values (structural path relationships) and t-values (significance of structural path based on t-value) in brackets

A brand is what sticks to the roof of the customers’ mind. It’s memorable and it is what differentiates a product in the marketplace. Branding is an exercise in perception (50). The brand “signature” or “personality” is based on sound strategic thinking. Brand differentiation is an exercise to capture customers’ perception. It determines the way you want the audience to perceive your product (51), and it is the first step in successful branding (52). For example, a reputation for innovation enhances credibility among customers. In particular, experimental studies have shown that innovation has made the acceptance of new product offerings more possible. It also helps a firm to gain reputation if it causes customers to believe that it shows concerns for them (53).

For firms, therefore, a well- managed brand becomes an important instrument of differentiation creating competitive advantage (54, 55). Furthermore, the differentiation achieved through branding, constitutes a barrier to entry, by making it difficult for competitors to emulate the companies’ offerings (56, 57).

Keller (1993, 2003) expresses that consumers choose brands on the basis of perceived differentiation and here, differentiation means relevant and unique added values which match their needs more closely (57). This means that differentiation is a tool for customers to choose different products, services, and brands. At last, it provides firms with brand equity and strength (58, 59).

It is often mentioned by other scholars that brands need to be differentiated in order to be purchased, since consumers must have a reason (60).

In differentiation strategy, a firm seeks to be unique. It selects one or two attributes that many purchasers in an industry perceive as important.

Differentiation is the first step in building brands.

Differentiation can take many forms from the clear-cut physical or functional, through the less distinguishable (two kinds of a product), the barely noticeable, the emotional (a mood or aspiration), to the ‹distinguishing but irrelevant› (packaging color).

Moreover, at the end of this part, Kapferer expresses that differentiation makes it possible for firms to increase the brand’s relevance, enlarge its presence and its visibility, whether online, among distributors, or on the shelf, if applicable. This also increases sales (49).

*Methods*

*Conceptual Model and Hypotheses*

In this study, the main construct is the uses of corporate reputation. This construct has three dimensions: value creation, strategic resources and corporate communication. Each of these three dimensions includes several items and is hypothesized to be related to brand differentiation which is utilized as an output of the uses of corporate reputation in this model.

This framework is developed by summarizing and synthesizing the works of a number of scholars (26, 36, 61, 62). who have previously studied the uses of corporate reputation. Chen-Chu Chen, (1) has suggested a model and we have extended her work by paying explicit attention to the influences of brand differentiation and company reputation.

As a matter of fact, in this study, we intend to investigate the impact of corporate reputation on brand differentiation-setting among brand managers and those who are effective in decision making for branding procedures in the Iranian pharmaceutical industry.

According to what we mentioned above, our hypotheses are defined as follows:

*H1:* *Value creation as a dimension of the uses of corporate reputation has a positive impact on a firm’s brand differentiation strategy.*

*H2: Corporate communication as a dimension of corporate reputation has a positive impact on a firm’s brand differentiation strategy.*

*H3: Strategic resources as a dimension of the uses of corporate reputation has a positive impact on a firm’s brand differentiation strategy.*

This research is a cross-sectional study and in terms of objective, it is an applied study and has used both qualitative and quantitative analyses, which are explained thoroughly in following subsections.

*Research Strategy*

The current study employed a “mixed method approach” which refers to the traditional view that quantitative and qualitative research might be combined to triangulate findings in order that they may be mutually corroborated (63). It employs collecting and analyzing data by both forms of research styles, qualitative and quantitative (64). The merit of this method is the fact that a qualitative study will excel at expressing the story, understanding complex social phenomena and assist the researcher in developing themes from the respondents’ point of view, while quantitative research will summarize a large amount of data for generalization purposes.

At the first phase of the study, a qualitative method is adopted (86), using content analysis of managers’ opinions on their decisions regarding brand differentiation strategy and the importance of corporate reputation uses for the pharmaceutical industry. 

This method is previously adopted by several researchers (1, 40, 65, and 66) to achieve the same objectives for corporate reputation studies.

At the sec phase, SCM (structural equation modeling) is applied to analyze the proposed model and to test hypotheses by using P.L.S. 2.0 software.

*Research Design*

*Qualitative Data Collection*

In order to make a qualitative data collection, after reviewing literature, semi–structured interviews were conducted to unfold what surrounds our phenomenon (67) as follows:

At First, a list of questions was designed on basis of the reviewed literature and the research question, along with open-ended questions (see Table 1).

After this step, a research framework was designed and provided to the interviewees.

Finally, the interviewees answered the semi-structured interview questionnaires so that a better perspective on the relationship between the hypotheses and related issues would be reached.

The number of interviewees was 18, which currently are working in pharmaceutical factories and companies as managing director, sales and marketing manager, branding manager, R&D manager, and responsible pharmacist.

*Research Setting*

Reviewing the literature shows that the majority of studies concerning corporate reputation and brand differentiation strategy have been conducted in western countries (the USA, the UK, Germany, Australia, and the Netherlands, etc.), which have limited any generalizability of theory (68, 69).

In order to bridge this gap, we decided to choose Iran, one of the most important countries in the Middle East as the setting of this study and pharmaceutical industry as one of the most important industries in Iran.

*Scale Development and Validation*

In this study our scale development procedure included three major steps:

The first step involves specifying operational definitions and dimensions of focal constructs to help the subsequent generation of hypothesized items to refer to each dimension. A literature search helped to achieve this step.

The sec step involves creating additional measurement items using semi-structured interviews with experts. The experts’ interviews included showing the conceptual framework to respondents and asking questions concerning the measurement items of each construct.

Before the final questionnaires were completed, respondents were asked to point out any item that was either ambiguous or difficult to answer (70). Subsequently, Cranach’s Alpha coefficients and item-to-total correlations were computed to check the reliability of measurement scales. Item-to-total correlations above 0.3 and Cranach’s Alpha coefficients above 0.7 were accepted as reliable scales (71, 72).

A set of questionnaires along with purified items from this step was edited and prepared for the main survey (73, 74). The final reliable and validated questionnaire which was ready to be distributed had measures and items as follows:

1_Value creation as an independent factor involved 10 questions.

2_Corporate communication as an independent factor involved 6 questions.

3_Strategic resource as an independent factor involved 7 questions.

4_Brand differentiation as a dependent factor involved 5 questions.

In the fourth step, following the main survey, purified measurement scales were tested if they could satisfy the hypotheses and sent to confirmatory factor analysis (CFA) as a method to confirm the scales. This procedure was employed to examine scale properties, such as reliability, and construct validity.

*Main Survey*

*Targeted Respondents and Sample Size*

The targeted participants of the main survey were managers and executives (managing directors, marketing managers, sales managers, general managers and their executives and responsible pharmacists) from the pharmaceutical industry in Iran. The respondents had enough knowledge and experience in setting brand strategies, which is related to corporate reputation.

Researchers use confirmatory factor analysis (CFA) to finalize the scales (72). A minimal sample size for CFA is usually recommended to be more than the number of co-variances in the input data matrix (75, 76). Since it has planned to use PLS to perform CFA, an empirical ratio of at least five observations per parameter has also been proposed (77). Based on the above discussions, and the number of experts who accepted to reply the questionnaires, the sample size in this study was 258. The number of repliers was 243 and 239 questionnaires that were valid.

## Results

*Reliability and Validity*

The results of confirmatory factor analysis are shown in Table 2, 3, 4, and 5

*Model Evaluation*

Structural equation modeling using PLS was used to evaluate the model. PLS (Partial Least Squares) method was used to test the hypothesized relationship between the research constructs as postulated in the conceptual model, and to assess the overall goodness-of-fit between the proposed model and the collected data set. In addition, a CFA was conducted by the software.

To test the model’s reliability, Cranach’s alpha coefficient is calculated. At first, it is calculated for the questionnaire using the SPSS software and the coefficient was 0.89, which is acceptable, and for each relation in the model alpha is as follows:

The Cronbach’s alpha coefficient for the relation between value creation and brand differentiation strategy, is 0.91, and between corporate communication and brand differentiation strategy, is 0.88, and between strategic resources and brand differentiation strategy, is 0.93.

For model validity, the CV Red. and CV Com. have positive amounts which show suitable validity of the model.

*Results of Test of Hypotheses*

Figure 3 shows the details concerning the parameter estimates for the model and results of the hypotheses tests are provided in Table 6.

The results provide support for the primary hypotheses so that corporate reputation has a positive influence on the brand differentiation strategy. The results also support the hypothesis (H1) strongly that value creation has a positive impact on brand differentiation (Path coefficient = 0.360, t_ value =3.167, *P* ≤0.05).

The results support the hypothesis (H2) that corporate communication has a positive impact on brand differentiation, but the impact is not particularly strong (Path coefficient = 0.022, 

t_ value =3.668, *p *≤0.05).

The results also support the hypothesis (H3) strongly that strategic resource has a positive impact on brand differentiation (Path coefficient = 0.289, t_ value =2.247, *p* ≤0.05).

The RSq of the model is 0.819 that supports the model considerably.

## Discussion

Brand management is getting the most important capability for several industries to differentiate companies from their competitors. According to Dannenberg and Kleinhans (2004), value creation occupies an important part of the brand management in a company (78). Furthermore, according to Lynch and Chernatony (2004), emotional brand value development may also cause value creation for their customers that can be a means of developing a sustainable differential advantage (79).

The researchers, in this study, assessed the relative influence of the all types of the uses of corporate reputation on brand differentiation by comparing their path coefficients and found the direct effects of all types of the uses of corporate reputation on brand differentiation to be positive and statistically significant.

In this case, the path coefficient of value creation (β = 0.360) and strategic resources (β = 0.289) differs hugely from that of corporate communication (β = 0.022). This indicates higher importance for value creation and strategic resources than the corporate communication on the brand differentiation. It means that in the Iranian pharmaceutical industry, brand differentiation depends on value creation and strategic resources more than corporate communication. In this study, it means that companies in Iran differentiate themselves and their products from competitors by focusing on their internal capabilities more than by negotiating with their stakeholders and customers

The results of this study are the same as what Holsapple and Singh (2001), Lynch and de Chernatony (2004), and Harrington (2007) had assessed before.

They asserted that pharmaceutical company managers use value creation, corporate communication, and strategic resources to implement their brand differentiation strategy to reach their targets more easily (79, 80, 81).

Mehralian, *et al*. (2011) asserted that the Iranian domestic pharmaceutical industry had not yet adequately developed to its full capacity and there are many potential capabilities for further growth and development (82). The researchers, here, suppose that by using the results of this study, the Iranian pharmaceutical companies can enhance their capacity and gain more advantages.

*Implications*

This study was motivated by the need for research that leads to a better understanding of the influences of branding and company reputation in pharmaceutical business markets.

In terms of methodology, the contribution of this research is two-fold. First, we tested reputation and branding models in a country outside of the United States and Europe owing to the necessity for cross-cultural research (21, 83, ,84) to establish the western researcher’s external validity of theories (68, 85). Sec, this study verifies, adapts and purifies existing measurement instruments in a country which is culturally different from the setting in which these items were first developed.

Finally this study enhances existing knowledge in branding and strategic management of medicines in countries like Iran.

In its strategic management view, this research shows that the concept of brand differentiation strategy can complement the resource-based view in explaining how it qualifies as a source of intangible assets and competitive advantages.

*Future Researches*

In this research, we have tested some western theories in a country of the Middle East. Maybe the same research in another country directs to another result, as Chen-Chu Chen (2011) did and had some different results, therefore we suggest to conduct the same research in another country particularly in the Middle East.

Another future direction of this research would be to develop a measurement to measure the relationship between this current study and financial performance to observe the impact of medicine price on the relationship of corporate reputation and financial performance of pharmaceutical companies in Iran or another country.

This study is applied to one industry, it would be significantly different for other studies which compare more than two industries in Iran or compare the same (pharmaceutical) industry in more than two countries.
